# Physical Effects of Maternal Deaths on Midwives' Health: A Qualitative Approach

**DOI:** 10.1155/2020/2606798

**Published:** 2020-04-01

**Authors:** Anita Fafa Dartey, Ellemes Phuma-Ngaiyaye

**Affiliations:** ^1^School of Nursing and Midwifery, University of Health and Allied Sciences, Ho, PMB 31, Volta Region, Ghana; ^2^Department of Nursing & Midwifery, Mzuzu University, Malawi

## Abstract

Grief does not only affect human emotions but also impacts their physical health. Understanding physical grief of people can bring to bear the grip of its daunting nature, a situation where routines become challenging. A qualitative explorative descriptive research method was used. A purposive sample of 18 ward supervisors and 39 ward midwives was used to ascertain the physical effects of maternal deaths on these caregivers in the Ashanti Region of Ghana. Data were collected through semistructured and focus group discussions. Data analysis was done parallel with data collection till saturation was reached. Ethics was obtained from the University of the Western Cape, South Africa, and Ghana Health Service. The findings indicated that generally, as a result of grieving over the deaths of their patients, midwives experienced physical health sufferings. Therefore, reported depression is expressed as insomnia, appetite loss, exhaustion, and social isolation. There is the need to reduce the physical effects of patients' death on caregivers in Ghana and therefore, the study recommends that all hospitals in Ghana utilize employee assistance programmes, a workplace intervention programme designed for such purposes.

## 1. Introduction and Background

Maternal mortality, a public health issue in Ghana, refers to the death of a woman during pregnancy or childbirth or within 42 days after delivery [1]. While it may appear as a single event, it bears catastrophic consequences on the emotional make-up of caregiving midwives charged with the responsibility of the well-being of pregnant women and their babies [2]. Midwives' regular attempts to deal with issues of maternal deaths in their work environments often translate into visible signs of caregiver depression, feelings of seclusion, and apprehension as well as being easily bothered, sometimes resulting in loss of sleep and appetite [3, 4].

Caregiver depression is a disturbance disposition that results from hassles associated with nursing care provided to patients. Depression is an important public health condition of general demonstrative dejection and withdrawal from people [5]. Depression creates a situation where people lose interest in normal and familiar activities [6]. In this state, the individual becomes moody and unhappy, and unwilling to do anything. This is likely because the individual wants to be by herself to think and reorganize her thoughts which are caused by feelings of depression, discouragement, worthlessness, and hopelessness for the future. Fuchs Thomas' research concludes that depression leads to lack of drive and “detunement” to work [6]. If not treated or taken seriously, this may lead to behavioural tendencies such as alcohol abuse, low self-esteem, absenteeism, chronic fatigue, and accident [7, 8]. Depression also is a risk factor for any cardiac-provoking situations as posited by Whang and colleagues [9].

In similar regards, sleep deficiency or deprivation becomes evident in the lives of caregivers who are traumatized physically by maternal deaths. This characterization of difficulty having restorative sleep resultantly poses work and coping challenges and influences personal quality of life as it impacts the cognitive abilities of midwives, thereby affecting their functionality both at work and at home [10–12]. They sleep late at night, wake up too early, or unable to sleep at all. Sleep plays an important role in the life of human beings; it refreshes the individual and gives the required rest [13, 14]. It creates stability in physical and cardiovascular health, and cognition. Continuous lack of sleep can lead to poor quality of life at the risks of hypertension, obesity, and heart disease. In extreme cases, nurses and midwives break down with exhaustion from emotional, physical, and mental episodes consequential of losing a patient to perinatal events [15].

In the African region, the paucity of literature on maternal death events, its consequences, and related effects on maternal health care somewhat plays to lessen the urgency to address issues of perinatal events among midwives. Furthermore, there are no studies done in Ghana to assess the physical impact of maternal deaths among midwives. Thus, this research intended to identify the physical burdens of maternal deaths, events on caregiving midwives in the Ashanti Region of Ghana.

## 2. Research Design and Methods

The study adopted a qualitative approach which is an interpretative methodological approach that helps create more subjective knowledge [16]. It develops from the behavioural and social sciences as a process of understanding the exceptional self-motivated and all-inclusive nature of human beings as sought by the study [17]. An explorative descriptive research design was used. The exploratory design targeted the discovery of new ideas and clarification of existing concepts. The use of exploratory research design helps the researcher to become familiar with basic details and become grounded for the situation under investigation, as it involves small groups of people. The descriptive design helped the researcher to bring out the meaning of the problem under investigation through a detailed description of the lived experiences of midwives at their workplaces. However, in doing this, the researcher had no intention determining the cause-and-effect relationship of the phenomena under investigation [18].

The study took place in the Ashanti Region of the republic of Ghana mainly due to the high number of maternal deaths recorded in the country. The Ghana Health Service 2016 annual report informs that the Ashanti region has 18% of total health-care workers in Ghana [19]. Thus, the recorded maternal mortality rate is 139 in 2014, 168 in 2015, 129 in 2016, 162 in 2017, and 221 in 2018 [20], figures higher than World Health Organization (WHO) Central and Eastern European regions with not more than the rate of 25 in the years under review [18]. The research study was conducted in nine health facilities including the teaching hospital, one regional referral hospital, four district referral hospitals, and three health centres. These facilities provide maternal health-care services with the type of service dependent on the level of each of the health facilities.

Purposive sampling was used because the data needed for the study can only be provided by a particular population (i.e., midwives), knowledgeable in the area of study or have experienced the phenomenon under investigation [21]. The sample size was determined by saturation of data. As a result, 39 participants formed eight (8) focus group discussions with four to seven participants. A focus group guide was used to facilitate the discussions, while an interview guide directed the semistructured interviews. Eighteen (18) participants took part in the individual semistructured interviews.

All the participants preferred to be interviewed at their work places. Both the interviews and focus group discussions began in the form of a social conversation and proceeded steadily to become highly interactive. Probing questions were used when appropriate to enhance the richness of data. The researchers used field notes to capture body language and facial expression of the interviewees. Each interview lasted between 30 and 40 minutes. The interviews were digitally recorded, checked for quality, and transcribed, and key findings were discussed within 24 hours.

## 3. Data Management and Analysis

The researcher used a letter and a number to code the interview schedule, while the same code was used to identify the individual informant's audio recording. Each of the respondents was coded as M1, M2, FG1M1, FG1M2, and so on. The interviews and focus group discussions were immediately transcribed verbatim in order to refrain from missing relevant data. A follow-up was held to review and verify the transcripts with the informants the following day. No major changes were requested by the informants. Thematic content analysis was used to process the transcribed data. The process included familiarization, identification of thematic framework, indexing, charting, mapping, and interpretation [22]. The researchers developed a coding scheme in which the theme and subthemes were labelled, categorized, and summarized, followed by charting, which involved rearranging the data within subthemes. The emerged subthemes were organized and interpreted to draw relationships between codes to aid easy presentation.

## 4. Trustworthiness

The trustworthiness of the study was achieved through audit trails. The researcher recorded events associated with the study over time and documented all the processes of the study, which can easily be followed by anyone interested and still obtain similar results. Self-reflexivity was ensured where the researcher reflected and examined the assumptions made in the study. These assumptions were made with regard to the data analysis, interpretation of data, and immersing oneself in the data analysis process so the researcher can be knowledgeable about the midwives' situation.

To ensure dependability, the coding process was evaluated at different phases by an independent coder. Neutrality was ensured through the strategy of conformability by keeping appropriate distance between the researchers and informants to avoid influencing the findings.

## 5. Ethical Considerations

Ethical clearance was granted by the Senate Research Committee of the University of the Western Cape and the Ministry of Health/Ghana Health Service. Additional permission was obtained from the Ethical Clearance Ashanti Region where the study was conducted. All the participants gave consent to take part in the study. Participants' confidentiality and privacy were maintained throughout the study by the use of alphabets and numbers in place of participants' name (e.g., focus group 1 midwife 1 (FG1M1) and individual midwife 1 (M1).

## 6. Findings

### 6.1. Demographic Characteristics of Respondents

All participants in the study were females, ranging from the staff midwife to the director of midwifery. Approximately 53% of the participants were from the junior-ranked officers. Midwives were between the ages of 22 and 61 years with 56.1% of them less than 46 years. This means that more young midwives suffer maternal death in their work life with coping challenges. The midwives had at least 3 years of work experience and at most 25 years of working experience since their first appointment. More than half of the participants have been in service for more than 10 years, giving indication that the midwives have good working experiences and possible multiple exposures to maternal death and the process that follows.

The key findings demonstrate the physical effects of maternal deaths on midwives working at the various hospitals in the Ashanti Region. The participants of the study perceived that their lives are physically influenced by the deaths of the patients they care for. Five themes were recognised, and these are depression, insomnia, loss of appetite, exhaustion, and social isolation. From observation, these effects were more prominent while participants were at home, where nothing is official (no professional ethics) and there was no need to pretend. Depression was seen as having influence on all the other themes.

### 6.2. Depression

Most of the participants, both in focus group discussions and the semistructured interviews, described some form of depression. The signs and symptoms of depression are very prominent among midwives when they experienced maternal death while on duty at the health facilities. Depression is a psychological state of mind but is observed through signs, symptoms, and changes in behaviour of a person who suffers it, resulting from exposure to psychic trauma like maternal death events [23, 24], as presented by quotes from the participants:

FG4M3: Yes, maternal death affects me so much; you feel bad all day and when you get home the family knows that there is something wrong with you because when you go home and you are moody, unhappy not feel like doing anything. M1: Your family members will see and feel that something is wrong with you.

Cases of social isolation emerged importantly in the data. Participants realized that when they experienced maternal death in the ward, they want to be left alone. They do not want to interact with other people, not even their own children. Some participants in this study prefer to be left alone without communicating with anyone around:

FG6M2: When I experience maternal death, I go home thinking about what went wrong, what did not I do right, why did this happen to me. FG6M2: I always think about it and am preoccupied with your thoughts. FG6M2: So it affects the relationship I have with other people because I am bored, so I isolate myself from people. FG4M3: When you experience maternal death and you go home, you do not want to speak to anybody; neither do you feel like doing anything with anyone.

Most participants reported the inability to observe personal hygiene such as taking their bath, neither are they interested in talking to family members when they get home. They rather go straight to bed:

FG5M4: For me, when I experienced maternal death and I got home; I do not even take a bath. I do not want to talk to anyone. I go straight to bed. FG5M4: When my children ask me what was wrong, I say nothing. FG4M1: Maternal death affects me in every way; chatting with people. I do not want to do anything or interested in anything. I want to be by myself.

### 6.3. Insomnia

The participants in the study demonstrated that insomnia was common among them when they experienced maternal death. The participants mentioned that they were preoccupied with the thought of the death of the deceased person. Lack of sleep is illustrated in the following quotes by the participants:

M13: I was so disturbed … that night because I was still thinking about maternal death that I could not sleep. M13: A client dying in your hands is not easy, you cannot sleep. FG3M2: Personally, my sleeping habits changed in such a way that I am not able to sleep. FG3M2: Am preoccupied by the thought of the death of the client. FG5M5: My mind is stress up, am not able to get sleep in the night. FG3M2: It affects my personal life…, my sleeping habits and so on.

### 6.4. Loss of Appetite

Participants observed that whenever they experienced maternal death in the discharge of duty, they are not able to eat. They explained that the event makes them lose appetite for food:

M17: Sometimes, when you go home, you cannot even eat because you feel like, what if it was you who has died, what would you do? Will you be eating? FG1M1: When a client I take care of died on the ward, sometimes if I go home, I cannot even eat to my satisfaction. M1: It will even affect the way you eat. This is because you will be thinking of maternal death. M8: Personally for me, when maternal death occurs at my ward, I feel very bad that day. At times I cannot even eat. FG8M3: When a mother comes in labour and then dies, you do not feel comfortable. Sometimes, when you go home, you cannot even eat. FG4M3: I do not feel like eating my food.

### 6.5. Exhaustion

Participants revealed that they get exhausted when they experience maternal death in the wards. They maintained that the exhaustion they feel makes it difficult to continue working, especially during the same shift. Exhaustion comes in when normal daily and simple activities become daunting because there is a lack of energy and interest in performing those activities as a result of the experience of maternal death:

FG1M4: From the moment that I experience maternal death, I cannot even do anything, even simple activities because it affects my emotions and I feel exhausted. FG5M3: Personally, the enthusiasm you use to work with goes away, exhaustion sets in.

## 7. Discussion

The participants in this study established that they were depressed in the face of maternal deaths, regardless of age, type of health facility they are working in, their rank, or work experience. Depression experienced by the participants in this current study relates closely to loss of interest in the environment or the people, as well as lack of self-care, neglect of family responsibilities, and inability to work. According to the participants, they do not like to talk to anyone; they just want to be by themselves. Some participants admit that they are not able to observe their everyday personal hygiene, difficulty keeping to routine of two baths a day, with possible trend in other hygiene practices; neither are they able to perform their responsibilities at home or work because their motivation and drive are cast off [25]. The participants in the current study fail to care for themselves when they experience maternal death in the health facilities. A midwife, who attends to a client in the hospital and the client unfortunately passes away, goes home and will not take a bath or clean her body considering that the amount of energy exerted during the emergency situation is grievous. Foureur, Besley, Burton, Yu, and Crisp [26] agree that self-care becomes a challenge to some nurses and midwives under this umbrella of stress.

Mark and Smith, Mark and Smith, Khamisa, Peltzer, and Oldenburg [27–29] acknowledged that depression is the most adverse emotional problems faced by nurses and midwives at their work places. According to the literature, depression among nurses and midwives is well known, but the needed support services are not standardized. This is in line with [30] who identified that depression causes poor mental health and thus leads to low productivity. These authors further explained that workers in occupations such as nursing and midwifery, teaching, and social work require support services to help them deal with depression.

In the results of this study are indications that participants in this study were unable to sleep when maternal death occurs at the hospital. Sleeplessness was associated with the mind, thinking about the incidence of death starting from when the client came into contact with them. This to the midwife is stressful because of the unrest of the mind. The state of sleeplessness conditions individuals with the inability to fall asleep or stay asleep. Poor quality of work life and poor quality of service delivery compromise client care standards gradually creating enabling environments for medical errors and avoidable clinical errors. The finding of the study agrees with related research, which found that stress affects sleep and results in loss of concentration, which leads to depression and lack of motivation to work [31–33]. The effect of psychosocial hazards to the midwives may affect their sleeping pattern, ability to concentrate, and motivation to work. Coping abilities of midwives are challenged by this result, risking further mental illness as associated with depression [34].

The participants in this study also alluded to the fact that they are unable to eat well after they experience maternal deaths. Eating is normal and an everyday activity. We eat to replenish our energies, to work, and live healthy lives. In this study, loss of appetite was eminent in most participants' data. Participants expressed their frustrations about having reduced desires to eat. They do not feel like eating anything because they are not hungry. Some emphasized that not only because they are not able to cook or access the food they want to eat, they are also not able to eat when the food is served. Within time, a person who experiences appetite loss soon may have related symptoms of weight loss, weakness, malnutrition, and immune competence. The consequential effects of these render affecting midwives physically unfit to satisfy the full demands of care as reduced energy levels subject them to fatigue with associated exhaustion. Prolonged loss of appetite may cause deficiency in important food nutrients that keep, protect, and grow the human body. A malnourished midwife becomes susceptible to sickness in the highly infectious environments of the facility as her immune system may not be able to fight off all infections. In this position, midwives are unable to contribute effectively to their collective caregiving efforts to pregnant women in providing safe motherhood for them. Torres and Nowson [35] are in line with this study, saying that stress affects eating behaviours of people. Though research [36, 37] reports that depression or stress also induces obesity through emotional eating, loss of appetite by same variable is a likely short-term effect [23]. What is often treated as superficial can be serious if left untreated. This less immediate consequences can bring to bear on maternal health-care practice in Ghana and the world.

Further, the results of the study identified that participants were found exhausted when maternal death happens in the wards. Participants observed that there were emergency situations before the deaths of their patients, in most cases. Some participants expressed frustration by indicating they did all they could to save the client's life but was not successful and the thought of it caused some form of exhaustion to their bodies. The experience of exhaustion felt by participants in the current study is attributed to the sudden loss of energy resulted from metal fatigue. Preoccupied memories, bargaining, and the mental stress of dealing with maternal deaths create a distaste for the job in midwives (emotional exhaustion) and directly impact their ability to work negatively. Be as it may be, exhaustion may also result from reductions in energy level associated with loss of appetite and malnutrition. Midwives who experience maternal deaths unconsciously deny themselves food. Their body and muscles are constantly not replenished with energy they used up working, so they are always weak and physically exhausted. This is in agreement with the findings of Houck and Hottensen [38, 39], who acknowledged that total exhaustion is one most common physical features of someone who has experienced death of another person. The experience of exhaustion does not only affect the work but also things that the participants do for herself and her family. For example, refusing to take a bath after nursing work or inability to perform maternal duties at home could pose an instability to her social relationships with likely effects on her mental well-being. Adwan [40] also concurs with the current study by her finding that caregivers such as midwives experience exhaustion when they lose patients under their care.

## 8. Conclusion

Overall, this study reveals the physical burdens of midwives in the Ashanti Region of Ghana that are made visible by behaviours emanating from emotional and psychological symptoms of grief associated with the experience of maternal deaths. Among them are visible signs of depression, impressions of insomnia, and loss of appetite, as well as crying, exhaustion, and frustration. Collectively, they impact the effectiveness of maternal health care given by midwives across the lengths of the Ashanti Region of Ghana and may in the process incur avoidable costs that plague maternal health-care delivery. In view of this, midwives need to be supported in varying capacities to enable them to cope comparably with the occupational hazards of maternal deaths and its adjoining outcomes. In addition, supervisors and all health-care providers should promote teamwork among midwives to address maternal deaths as aimed in the SDG 3.

Currently, counselors are available for staff who face work and family difficulties at the teaching hospital.

### 8.1. Limitations of the Study

The study cannot be generalised since it was qualitative and the views of participants are mainly subjective.

## Data Availability

This study is part of a PhD work on “The development of an employee assistance program for midwives dealing with maternal deaths”. Hence the data can only be assessed by clearance from the Department of Nursing, University of Western Cape, South Africa.
